# Patient uptake and adherence to social prescribing: a qualitative study

**DOI:** 10.3399/bjgpopen18X101598

**Published:** 2018-08-08

**Authors:** Julia Pescheny, Gurch Randhawa, Yannis Pappas

**Affiliations:** 1 PhD Student, Institute for Health Research, University of Bedfordshire, Luton, UK; 2 Professor of Diversity in Public Health and Director, Institute for Health Research, University of Bedfordshire, Luton, UK; 3 Head of PhD School and Reader in Health Service Organisation and Delivery, Institute for Health Research, University of Bedfordshire, Luton, UK

**Keywords:** Integrated care, primary health care, social prescribing, uptake, adherence

## Abstract

**Background:**

Social prescription is an initiative that aims to link patients in primary care with sources of support within the community and voluntary sector to improve their health, wellbeing, and care experience. Such programmes usually include navigators, who work with referred patients and issue onward referrals to sources of non-medical support. Most research on social prescribing (SP) has focused on outcome evaluations, resulting in a knowledge gap of factors affecting uptake and adherence. Understanding such factors enables the refinement of programmes, which has the potential to enhance uptake and adherence, reduce health inequalities, and optimise investment.

**Aim:**

To explore the experiences and views of service users, involved GPs, and navigators on factors influencing uptake and adherence to SP.

**Design & setting:**

Qualitative interviews were conducted with stakeholders involved in an SP programme in the east of England (Luton).

**Method:**

Data were collected from semi-structured face-to-face interviews with service users, navigators, and GPs. Thematic analysis was used to analyse the data.

**Results:**

Factors affecting uptake and adherence to SP were related to patients’ trust in GPs, navigators' initial phone call, supportive navigators and service providers, free services, and perceived need and benefits. Reported barriers to uptake and adherence were fear of stigma of psychosocial problems, patient expectations, and the short-term nature of the programme.

**Conclusion:**

This study provides an insight into factors affecting patient uptake and adherence to SP programmes. More research in this field, including patients who refused to participate in SP, is needed.

## How this fits in

Primary care patients present both medical and psychosocial problems to GPs. Responding to psychosocial problems, such as social isolation and housing issues, is often beyond the capacities of healthcare professionals and too complex to address within the time constraints of a consultation. SP provides healthcare professionals with a referral option to address the psychosocial needs of patients more effectively. SP has the potential to improve the health and wellbeing of service users and to reduce health resource use; however, research on factors affecting uptake and service user adherence is limited.

## Introduction

Psychosocial problems, such as debt, housing concerns, social isolation, domestic abuse, family problems, grief, and loss, can impact on peoples’ mental and physical health, wellbeing, and self-care.^1–3^ Patients present both medical and psychosocial problems to GPs, who are the first point of contact to seek treatment and referrals in the NHS and other health systems globally.^4–7^ In order to maximise the health and wellbeing of patients, primary care professionals need to be able to respond to both patients’ medical and psychosocial needs.^7^ However, due to time pressure and the lack of available resources, responding to psychosocial problems such as housing issues or social isolation can be a frustrating experience for GPs.^8,9^ Research found that GPs rarely refer patients to local community groups or advice services, due to a lack of up-to-date knowledge of local resources.^5^ GPs tend to respond to psychosocial problems with consolation and reassurance, or treat the consequences of psychosocial problems with medical interventions, rather than responding to the psychosocial problems themselves.^8–10^ In general, available sources of non-medical support within the third sector to address psychosocial problems of patients remain underused, due to weak or non-existing links between health services and organisations in the third sector.^11^ Expanding the referral options of primary care practitioners to non-medical support could save professionals’ time, increase their satisfaction, enhance the sustainability of general practice, and address the psychosocial problems of patients more effectively, which are often the source of poor mental and physical health and wellbeing.^8,12^

SP is an approach that provides GPs with a non-medical referral option to address the non-medical needs of patients, affecting their health and wellbeing. It is a way of linking patients in primary care with sources of non-medical support, typically provided by the third sector (including charities, voluntary, and community groups).^13^ Different SP models exist, including various referral routes (such as self-referral, referrals from community centres, or from healthcare professionals), pathways, and cooperation types across the third and health sectors.^6,14,15^ Recognising that simply giving information to patients about services results in low uptake, most SP schemes involve a navigator to provide personal support.^9^ In such models, the role of navigators can be specifically developed as part of the intervention or performed by volunteers, health workers, or development workers.^6,15–17^ Typically, the role of navigators involves: individual assessment to identify non-medical needs of service users and motivational interviewing; help to access non-medical sources of support; continuous personalised support; and data collection for evaluations.^17^ Examples of sources of support, predominantly provided by local services, include art therapy, walking and reading groups, exercise classes, nature-based activities, volunteering, legal advice, and support with employment, debt, and housing.^17^

Most of the research on SP focuses on outcome evaluations.^17–22^ Available research suggests that SP interventions have the potential to reduce demand on primary and secondary care and to improve feelings about health and wellbeing of service users, as well as health-related behaviours.^17,23,24^ General improvements in ability to carry out everyday activities, quality of life, and feelings of loneliness and social isolation were also observed.^17,23,24^ There is limited evidence on factors affecting initial participation of patients (uptake) and continuous participation (adherence) in SP programmes.^25^ The consequences of non-uptake and non-adherence may include preventable suffering, suboptimal outcomes, increases in health inequalities, and wasted resources.^26,27^ The identification of factors hindering uptake and adherence could support the refinement of SP programmes to enhance uptake and adherence, as well as optimising investment.^25,26^

**Figure 1:**
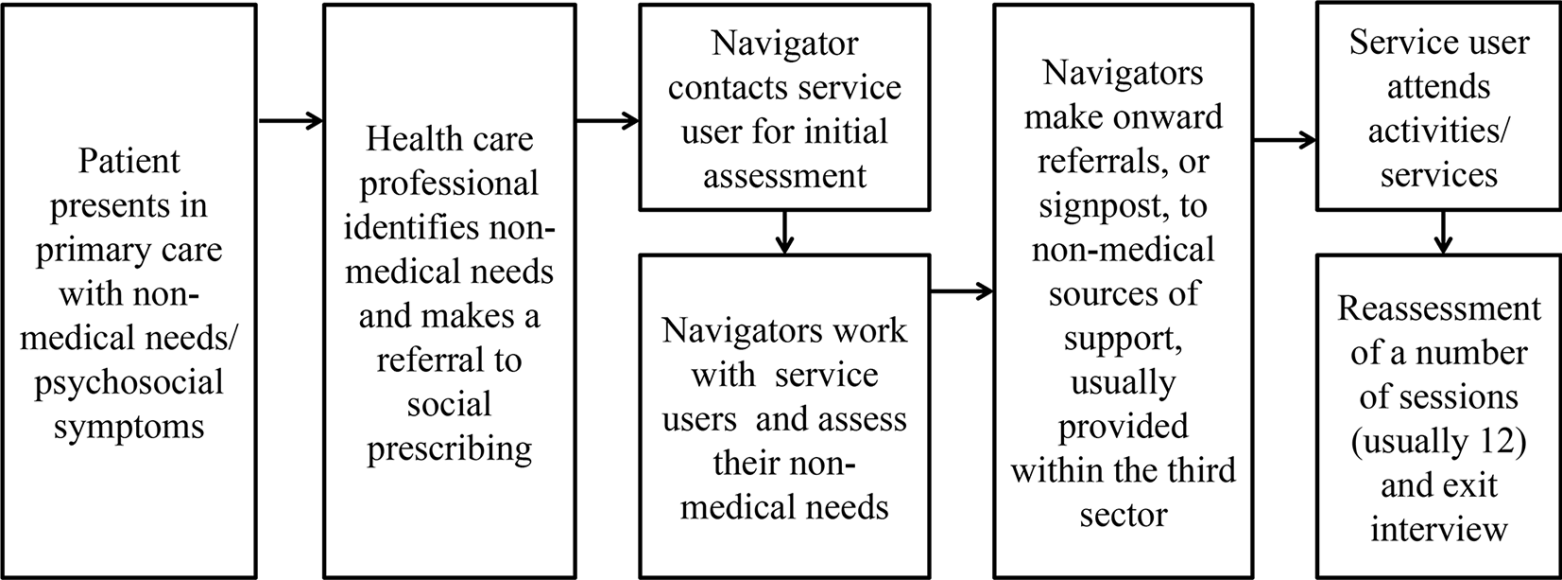
Luton social prescribing pathway

The aim of this study is to explore factors affecting users’ uptake and adherence to SP. In this study, uptake is defined as agreeing to be referred to SP and attending the first appointment with a navigator. Adherence is defined as continuous participation in navigator appointments and referred services. This study focuses on an SP programme, which was implemented in one Clinical Commissioning Group (CCG) area in the east of England (Luton) across four general practices. In the Luton model, the pathway starts with a referral from a healthcare professional to a navigator. Navigators were recruited and trained specifically for the intervention. Following referral, navigators contact primary care patients to arrange an initial appointment held in surgeries, and perform their role as described earlier. In the Luton SP model, navigators can refer service users onwards to a maximum of 12 sessions, which are free of charge for service users. In order to receive referrals from the social prescription programme, service providers in the third sector have to complete an accreditation process. The intensity of support provided by navigators depends on the service user’s needs and circumstances. The pathway of the Luton SP model is displayed in Figure 1. More information on the Luton SP programme is available elsewhere.^28^

## Method

This study is part of a larger mixed methods doctorate study (2015–2018), focusing on the evaluation of the Luton SP programme as an example of the integration of social and health care.

### Setting

This study was set in the east of England in an area of high socioeconomic deprivation (Luton), ranked as the 59th most deprived area in England by the Index of Multiple Deprivation (ranging from 1 [most deprived area] to 32 844 [least deprived area]).^29,30^

### Study design

Given the study’s focus on the experiences and views of service users, navigators, and GPs, a qualitative approach was employed. The study used face-to-face semi-structured interviews with GPs and navigators involved with the local SP programme and service users across various engagement levels. Data were collected from November 2016 to October 2017.

### Participants and recruitment

All employed navigators (*n* = 4) and participating GPs were invited to the study via e-mail. The Luton CCG assisted the recruitment of GPs by emailing an invitation to the study to the eight lead GPs (across the four practices) and the four practice managers. The CCG also encouraged GPs and practice managers to share the email with partners within the practice. An information sheet explaining the purpose of the study and a contact form were attached to the e-mails. GPs and navigators were asked to return the contact form to the researcher to indicate whether they are interested in participating, and if so, their preferred communication method to arrange an appointment. Due to structural changes in the NHS, one participating surgery was closed at the beginning of the recruitment period of this study. The closure of this surgery required the transfer of patients to other participating surgeries. Besides several attempts and reminders, one GP practice refused to participate in the study, due to the increased workload of registering new patients. Several emails and reminders were sent to GPs and practice managers of the other three surgeries.

Navigators acted as gatekeepers and sent or handed out recruitment packs, consisting of an information sheet, a contact form, and a pre-paid envelope to eligible service users (Box 1) on behalf of the researcher. To ensure that consistent information is provided to service users and selection bias reduced, a recruitment guide was developed and discussed with navigators. In total, 30 recruitment packs were sent out to eligible service users and 16 packs were handed out to service users. High staff turnover and significant delays in recruiting and setting up new navigators hindered the recruitment of service users for this study. In addition, navigators reported that when introduced to the study, the majority of service users declined to participate. Service users who received the recruitment pack were asked, if interested in participation, to send the contact form back to the researcher, using the pre-paid envelope, or to return it to their general practice reception. The researcher contacted service users by telephone to ascertain willingness to participate and, if willing, to arrange an appointment.

**Box 1.** Inclusion and exclusion criteria for service users

Inclusion criteria	Exclusion criteria
· Referred to the social prescribing service by a GP · Referred to social prescribing some time over the past year (from the point at which recruitment of participants start in the study) · Sufficient English-speaking skills to take part in the study	· Service users with significant hearing impairments

### Data collection

Individual face-to-face semi-structured interviews were conducted with GPs, service users, and navigators. Interviews took place at the primary care surgeries and were audio-recorded and transcribed verbatim (range 30–75 minutes). Due to the different experiences and roles of the three stakeholder groups, three interview schedules were developed on the basis of existing literature, the Luton SP pathway, and through discussion with the research team. The initially developed interview schedules were piloted with a convenience sample of stakeholders from each group.

### Data management and analysis

Transcripts were anonymised and checked against recordings for accuracy. Transcripts were uploaded to NVivio (version 11) and analysed employing thematic analysis.^31^ An inductive approach was employed to identify, analyse, and report patterns in the data. After the familiarisation with the data, key units of the texts, through the prism of the research question, were coded using line-by-line coding and the constant comparative method within and between transcripts.^32,33^ The inductively derived codes were organised into categories and themes, which were regularly discussed with the wider research team. Deviant analysis, where the researchers examined and reported opinions and experiences that contradict central interpretations, was used to enhance validity.^34^

## Results

A total of three GPs, two navigators, and 10 service users (Tables 1–3) were interviewed to explore the reasons for patient uptake and service user adherence to the SP programme.

**Table 1.** Service user sample characteristics, reasons for referral, type and number of referred services, and status at point of interview

ID	Sex	Employment status	Marital status	Ethnicity	Age	Reasons for referral	Type and number of referred services	Status (at point of interview)
Service user 1	Female	Unemployed	Married	White-British	56	Depression and anxiety, unemployed and needs help to find a new job, weight loss	Job centre support (1), physical activity (1), social activity (1), and sewing group (1)	In process: engaged with job centre appointments, engaging with physical activity, waiting to hear back from sewing group, planning to engage with social activity (not started at point of interview)
Service user 2	Female	Unemployed (cannot work due to health conditions)	Legally separated	White-British	59	Depression, targeted physical activity to support recovery, loneliness	Mental wellbeing services (1), support group (1), and physical activity (1)	Finished: engaged with mental health services, did not engage with physical activity
Service user 3	Female	Retired	Married	White-British	66	Depression, loneliness, sedentary lifestyle	Art class (1) and physical activity (1)	In process: engaged with art class, dropped-out of physical activity
Service user 4	Female	Retired	Married	White-British	64	Carer, poor stress management skills	Meditation (1), physical activity (1), massage therapy (1)	Finished: dropped out of meditation, did not engage with physical activity, engaged with massage therapy
Service user 5	Female	Unemployed (Homemaker)	Married	Egyptian	47	Back pain, loneliness	Massage therapy (1), community group (1)	Finished: engaged with massage therapy, did not engage with community group
Service user 6	Female	In full time employment	Married	White-British	39	Weight loss	Weight management (1)	Finished: did not engage with service
Service user 7	Female	Carer for mother and husband	Married	Pakistani	29	Weight loss, depression, stressed, carer	Physical activities (3) and mental health service (1)	In process: engaged with physical activities, waiting to hear back from mental health service
Service user 8	Female	Unemployed	Single	Pakistani	34	Stressed, mental health problems, back pain, functional barriers	Housing advice (1), social and family workers (1), legal advice (1), community service (1)	In process: engaged with advice and social services, did not engage with community services after first visit
Service user 9	Female	Student	Single	Black-British	27	Mental health problems, weight loss, housing problems, loneliness, functional barriers, issues with her son (toddler)	Physical activity (2), weight management (1), housing advice (1), community service (1), children centre (1)	In process: engaged with advice service, and one physical activity, waiting to hear back from weight management service, did not engage with one physical activity, community service, and children centre
Service user 10	Male	In full time employment	Married	White-British	54	Weight loss, depression	Physical activities (2), mental health service (1)	In process: engaged with physical activities and mental health service

**Table 2.** GP demographics

Characteristic	*n*
**Age, years**	
40–49	1
50–59	2
**Sex**	
Female	1
Male	2
**Working hours**	
Full time	2
Part time	1
**Type of GP**	
Salaried	1
Partner	2
Years of practice	
5–10	1
21–25	1
26–30	1

**Table 3.** Navigator demographics

Navigators	*n*
**Age, years**	
20–29	2
40–49	2
**Sex**	
Female	3
Male	1
**Ethnicity**	
White British	3
Mixed white and black African	1
**Working hours**	
Full time	4
Part time	0

### Uptake

#### Trust in GPs

In the Luton SP programme, primary care patients have to consent to be referred to the SP programme by their GPs. GPs, navigators, and service users have expressed the view that patients’ trust in GPs is an important factor promoting primary care patients’ uptake of the SP programme:

*'If, I mean, even if possibly another doctor would have recommended it, the thing is I know* [name of GP]*. We know each other for so long, I trust him. And I trust him that he knows me well enough, so I said "yeah okay."'* (Service user 3)

#### Perceived needs and benefits

Service users and GPs reported that recognising the potential benefit of SP and need for help to deal with non-clinical problems oneself drove primary care patients’ uptake:

*'One question was: "Do I need help?" And my answer was: "Definitely yes!" And it all sort of started from there really.'* (Service user 2)

Some service users emphasised that they have understood the potential benefits of SP for themselves, for example pain relief, reducing medication, and improving mental health conditions, after the GP has pointed them out. This indicates that good communication with referrers about the SP programme, and its potential benefits, plays a key role in patient uptake.

Service users contrasted social prescription favourably with what they perceived as the outcome of the use of relevant medication. Service users believed that, in contrast to medication that is prescribed to improve mental wellbeing, SP has the potential for a long-term benefit:

*'Well, I do not want to pop any more pills than I have to. I regard pills as a short-term solution and I thought this is something more than a short-term solution. I mean happy pills might get me through the winter, but what then?'* (Service user 3)

All interviewed GPs reported that those patients who refused to be referred to the SP programme believed that they don’t need SP and will not benefit from it:

*'I had a person who declined* […] *she was a carer for a* [man with dementia]*, she goes like: "Actually I have things to do, they take me out." She actually felt that she did not need to get involved because she is already doing enough other things and getting support from other areas, so she did not feel the need.'* (GP 1)

#### Free services while being enrolled in the SP programme

The free nature of the SP programme seemed to promote service user uptake:

*'I thought: "It is free, I try it.”'* (Service user 4)

However, a GP reported that some primary care patients refused to participate in the SP programme because of its short-term nature, meaning that only a limited number of free sessions were offered:

*'Patients asked: "How long can I go to the gym for free?" And I said: "Probably as long as you are on the programme, but the number of free sessions is limited, it’s about a couple of weeks." Then they are not interested anymore, they ask: "What is the point then if it’s not free anymore?'”* (GP 2)

#### Initial phone call from navigators

Following a primary care referral, navigators contact service users by telephone to arrange an initial appointment held in surgeries. Narratives indicated that this pathway promoted the uptake of patients who forgot about SP, or would not have made the first step to arrange an appointment to see the navigator:

'*Oh yes, I forgot about it and then she* [the navigator] *called.'* (Service user 4)

Some service users reported that they didn’t fully understand the SP programme when they were referred by their GP. Receiving a call from the navigator provided patients with an opportunity to ask for clarification, which seemed to promote their engagement with the first appointment:

*'I asked what this is actually for. And then she* [the navigator] *said all we do is sit there, we talk, I find out if there is anything I can help with, any introductions, any particular type of group or anything you know associations that I could get you involved with or introduce you to* […] *Yeah, and then I came here to the surgery.'* (Service user 2)

#### Patient expectations

As SP, a non-clinical intervention, is implemented into a clinical setting, largely based on the medical model of health care, it may not be aligned with the expectations of some patients:

*'Patients expect to be referred to an investigation, or a drug, not to SP!'* (GP 3)

GPs reported that a reason for non-uptake is that patients are entrenched in the traditional medical model, and expect a medical solution, such as a drug therapy, rather than actively engaging in an activity to address non-clinical needs affecting their health and wellbeing:

*'I suppose it is something unusual and it is not what you expect* […] *You know, you just want to go home, tell* [your family] *you got a prescription, and that you will be better in a week. And this is not like that. This is long-term, it is not a quick fix. It is more about whole lifestyle change and patients* [being] *actively involved. And that is why they refuse to become involved with SP.'* (GP 3)

#### Stigma of psychosocial problems

A GP reported that the perceived stigmatising attitudes of the society to patients with mental illness and social problems might work as a barrier to uptake:

*'* [...] *they* [patients] *are afraid of being stigmatised. They feel that in some way they have not created the social environment that maybe is best for their wellbeing.'* (GP 3)

This GP expressed the view that social and psychological determinants of health, which are addressed in SP programmes, are less accepted in the society than biological determinants of health:

*'* [...] *very often symptoms that have an underlying psychological or social basis, I think the society, sadly, you know, the stigma pervades the whole of society … and it is easier for people to acknowledge that you are ill if you have a physical label. And if you don’t have a physical label to your condition, you are viewed perhaps with not that much sympathy as others.'* (GP 3)

Thus, the stigma attached to the non-medical needs of patients may act as a barrier to the uptake of initiatives beyond the traditional biomedical model of healthcare.

### Adherence

#### Navigator approach

Service users emphasised that the navigators’ person-centred approach facilitated feelings of trust, control, and readiness to reflect on their current circumstances and their non-medical needs. Service users reported that they engaged with further navigator appointments because they felt listened to and valued. In addition, service users reported that appointments with navigators felt less rushed and, unlike with GPs, they felt able to discuss their non-clinical needs without being pointed to a medical solution to deal with the consequences of the non-medical problems:

*'Social needs, mental needs, you know, just general back-up in life in general. I never felt that I had that before this* […] *I was able to open up to* [navigator]. […] *If you go to the GP and you say "I am having trouble at home", they say "oh take this tablet or take this pill, oh you will be fine in a couple of weeks", or you know, "see what it is like and come back in a week time if it is not better". I am on enough tablets, the last thing I want is more tablets!'* (Service user 2)

Service users appreciated not feeling under pressure to agree to activities suggested by navigators, if they didn’t like them. Empowering and actively involving service users in decisions on their onward referrals facilitated feelings of control. Feeling in control and freedom to decide in which service to participate, promote service users’ adherence:

*'And I said that I also want something sort of that occupies my mind. And that is when she suggested the art class, which has been absolutely brilliant and exactly what I wanted* […] *and I explained on the physical side that I am severely limited. She printed out for me the gym programmes at the various health centres, so I could decide where I wanted to go.'* (Service user 3)

#### Navigator support

Service users repeatedly reported that the support of and work with the navigators facilitated the feelings of readiness to engage with services they would not have engaged with otherwise:

*'And she* [the navigator] *helped me quite a bit actually. She has been very good actually, I went to the gym this morning. There is no way I would have gone to the gym without her. No way I would have done it!'* (Service user 1)

The level of support that service users required in order to engage with services was dependent on their individual needs. Navigators believed that accompanying service users to first sessions, and helping them to build confidence, self-reliance, and eventually independence were crucial steps in determining the adherence of some service users:

*'* [...] *you have given them the confidence and you might even have helped them go by taking them and holding their hand, introducing them, be there for a couple of sessions with them.'* (Navigator 2)

#### Service provider support

Service users emphasised that feeling supported from service providers was a key factor determining their adherence to onward referrals. When service users haven’t received a response from service providers, they didn’t engage with referred services:

*'But I haven’t actually done anything, but only because no one would get back to me.'* (Service user 6)

On the other side, service users reported that knowing that a provider is awaiting them and supportive, for example picking them up from reception and welcoming them at the first session, boosted adherence to onward referrals:

*'Yes, so I knew someone was waiting for me* […] *Yes, I would just sit and waited at the table, and she picked me up.'* (Service user 1)

Ongoing support and motivation, especially during physical activities, were identified as another factor promoting service user adherence to the SP programme.

## Discussion

### Summary

This study identified factors determining uptake and adherence to an SP programme in Luton, UK. The fact that the referral to the SP programme was made by trusted GPs promoted the patients’ uptake of SP. In addition, programme design-related factors — such as a pathway in which navigators initiate the first contact with patients and free services — promoted uptake. Perceived need and benefits at the point of referral seemed to encourage uptake, too. Issues that appeared to hinder uptake include the short-term nature of the programme, patient expectations of primary care, and fear of stigma. This study found that a patient-centred approach and continuous support from the navigator and service providers promoted adherence.

### Strengths and limitations

The study sample included GPs responsible for referring patients to the SP programme, navigators contacting and working with service users throughout the programme, and service users across all engagement stages. Involving multiple stakeholder groups allowed the exploration of patient and service user engagement from different perspectives. As primary care patients who refused to get involved with SP could not be identified retrospectively, reasons for initial refusal from a patient perspective could not be studied. To compensate for this limitation, GPs were asked about patients’ reasons for refusal to get referred to SP. More female than male service users participated in the study, offering an unbalanced view, as women were overrepresented in the study sample. Another limitation is the low response rate from GPs and service users, which may be partly attributed to the structural and practical changes that happened during the recruitment period of this study. However, challenges associated with recruiting healthcare professionals for research studies were noted in previous research.^8,35^ Findings of this study are based on one SP programme in the UK, and therefore may be less relevant for different SP models and settings. Nevertheless, this study can provide initial insights and guidance for policymakers and providers planning to implement SP within primary care.

### Comparison with existing literature

In line with findings of previous studies in primary healthcare settings,^36–38^ this study found that a trusted relationship between patients and health professionals promotes acceptance of physicians' recommendations and improves adherence to health-related interventions due to patients’ trust in GPs’ judgments and advice. It is interesting that besides the reported trust, service users in this and other studies,^8,39^ reported difficulties in discussing their non-medical needs with GPs. Likewise, studies found that GPs find it troublesome and time-consuming to deal with patients’ non-medical problems, and therefore may be reluctant to probe for these.^4,8,9,39^ These findings suggest that navigators can have a vital role to play within primary care teams in the identification and response to non-medical needs of patients. Consistent with the findings of a systematic review^25^ and recent studies^17,40^ on predictors of primary care-based exercise referral scheme uptake and adherence, factors related to the programme design, including costs, methods to invite individuals, and navigator support, were identified as relevant factors. A direct invitation via telephone, including a specific day and time for the first appointment, as well as free services while being enrolled in a programme, seemed to promote uptake in this and previous studies.^25,40^ Although free services may promote uptake and adherence to SP, they may hinder long-term adherence to behaviour changes due to financial constraints to continue activities beyond the programme. This issue may be particularly relevant when SP is implemented in areas with high deprivation levels. Consistent with this study, Moffat *et al* and Killingback *et al* found that the navigators’ characteristics, support, and person-centred approach facilitated the uptake of services and ongoing adherence to SP. The present study is novel, however, in its involvement of GPs to explore the reasons for non-participation of primary care patients. Involving this stakeholder group, in contrast to enrolled service users only, allowed the identification of fear of stigma and medical expectations from primary healthcare as potential barriers to uptake. It is likely that the biomedical focus in primary care results in expectations of patients to be treated with medical interventions.^41,42^ Thus, improving the uptake of non-medical interventions in primary care may require a shift in societies’ perceptions towards a biopsychosocial model of healthcare, considering that health is influenced by biological, as well as social and psychological factors. Similarly to this study, studies found that individuals participated in community-based group exercise programmes because they perceived the need of remaining fit and well and believed in potential benefits, including losing weight, staying active, or maintaining their independence in daily life.^40,43^

### Implications for research

Considering the factors identified in this study when developing, or reviewing, an SP programme may help to promote the uptake of SP and enhance adherence.^25,26^

Given that patients who refused to be referred to SP were not involved in the current study, future research including this patient group is needed to improve communication with this group and to understand the reasons for non-uptake from a patient perspective.^44^ Furthermore, the authors recommend to collect data on uptake issues and sociodemographic characteristics of patients who refused to get involved, in order to reduce potential health inequalities^25,27^ and enable a more robust economic analyses of SP programmes.^25,45^ Although sample size is not central to qualitative research, future studies should aim to include more healthcare professionals and service users to explore their views and experiences in depth.^46^
